# Learning Through Retrieval Variability: Benefits and Metacognitive Judgments in Foreign-Language Vocabulary Learning

**DOI:** 10.3390/bs16091705

**Published:** 2026-09-21

**Authors:** Nicholas C. Soderstrom

**Affiliations:** Department of Psychology, Montana State University, P.O. Box 173440, Bozeman, MT 59717-3440, USA; nicholas.soderstrom@montana.edu

**Keywords:** variable practice, retrieval practice, studying, learning, metacognition

## Abstract

Variable practice often enhances long-term learning despite increasing difficulty during acquisition. The present study examined whether variable retrieval practice improves foreign-language vocabulary learning and, if so, whether learners’ metacognitive judgments reflect its benefits. Participants studied Lithuanian–English word pairs across four study–test cycles under either constant or variable retrieval practice conditions. In the constant conditions, learners consistently retrieved words in either an easy or difficult direction, whereas in the variable conditions, they retrieved in both directions. Following practice, participants predicted their performance on a final test, which was administered after either 10 min (Experiment 1a) or 24 h (Experiment 1b). Results showed that variable retrieval practice improved retention, with the clearest benefits observed when items were later tested in the more difficult retrieval direction after a substantial delay. Although learners’ predictions failed to anticipate these benefits, participants appeared to recognize the effectiveness of variable retrieval practice, and many reported using similar study strategies independently. The findings extend the literature on desirable difficulties and variable practice by demonstrating the benefits of variable retrieval practice in foreign-language learning while highlighting a disconnect between learning outcomes and prospective metacognitive judgments. Practically, the results suggest that incorporating variable retrieval practice into vocabulary instruction may enhance retention and promote more effective self-regulated study strategies.

## 1. Introduction

A major goal of educational and cognitive psychology is to identify learning conditions that promote durable and transferable knowledge. Although learners often prefer study strategies that make acquisition feel easy and fluent, a large body of research demonstrates that conditions that introduce challenges during acquisition frequently produce superior long-term retention and transfer. Bjork (1994) referred to such manipulations as desirable difficulties—learning conditions that impose short-term performance costs but enhance long-term learning outcomes. Examples include spacing study sessions over time (for a review, see Cepeda et al., 2006), interleaving different materials (for a review, see Firth et al., 2021), varying the conditions of practice (for a review, see Raviv et al., 2022), and engaging in retrieval practice (for reviews, see Roediger & Karpicke, 2006b; Rowland, 2014). Across these learning conditions, learners often perform worse during acquisition while ultimately demonstrating better retention and transfer (see Soderstrom & Bjork, 2015). Understanding why desirable difficulties improve learning and whether learners appreciate their benefits remains an important objective for both theory and practice.

One of the most robust desirable difficulties is retrieval practice. Rather than simply restudying information, learners benefit from actively attempting to retrieve previously studied material from memory. Retrieval practice improves long-term retention across a wide variety of educational materials (Carpenter & Pashler, 2007; Pan & Rickard, 2018; Roediger & Karpicke, 2006a; Soderstrom & Bjork, 2014). Importantly, retrieval practice can often create an illusion of poorer learning because successful retrieval is effortful and performance during practice can be relatively low. Consequently, learners may underestimate the benefits of retrieval practice despite its demonstrated effectiveness (Roediger & Karpicke, 2006a).

A related line of research suggests that introducing variability into the acquisition process can also enhance long-term learning. Variable practice has been examined extensively in motor learning, where practicing multiple variations of a task often leads to superior retention and transfer compared with repeating a single variation (for a review, see Guadagnoli & Lee, 2004; Schmidt, 1975). Similar benefits have also been observed with verbal materials, category learning, problem solving, and analogical reasoning (Gick & Holyoak, 1983; Goode et al., 2008; Paas & Van Merriënboer, 1994; Smith & Handy, 2014). Varying study conditions or practice schedules can lead to deeper processing and help learners encode a broader range of retrieval cues, which, in turn, can facilitate long-term retention and transfer (Estes, 1955). Like retrieval practice, however, variable practice frequently comes with a short-term cost by impairing immediate performance.

Although desirable difficulties have been extended to vocabulary learning (Bjork & Kroll, 2015), little research has examined whether retrieval variability benefits foreign-language vocabulary learning. Vocabulary acquisition provides an especially useful context for examining this question because retrieval difficulty can be manipulated naturally by reversing cue-target relationships. For example, recalling an English word from a foreign-language cue is generally easier than recalling a foreign-language word from an English cue. Indeed, Schneider et al. (2002) showed the learning benefits of retrieving foreign-language vocabulary pairs in the more difficult direction. Their participants studied and were given practice tests on French–English word pairs in either the easy direction (e.g., trying to recall *mouth* when given *bouche*) or the difficult direction (e.g., trying to recall *bouche* when given *mouth*). They found that long-term retention was enhanced if participants retrieved the pairs in the difficult direction compared to the easy direction, despite acquisition performance showing the opposite pattern. Importantly, although Schneider et al. varied item difficulty across participant groups, they did not include a condition in which participants practiced the pairs in *both* directions. In other words, their groups engaged in constant practice (either exclusively easy or exclusively difficult), not in variable practice.

In addition to identifying and understanding specific learning conditions that promote retention and transfer, it is important to determine whether learners recognize the benefits of those conditions. Metacognition refers to individuals’ knowledge and monitoring of their own cognitive processes (Flavell, 1979; Nelson, 1996). One of the most commonly studied metacognitive judgments is the judgment of learning (JOL), in which learners predict how likely they are to remember information on a future test (Rhodes, 2016). JOLs play an important role in self-regulated learning because they influence how learners allocate study time, select learning strategies, and evaluate their progress (Nelson & Narens, 1990). Ideally, learners would use accurate metacognitive judgments to guide their study decisions; however, research consistently demonstrates that JOLs are often influenced by cues that are only weakly related to actual learning outcomes (for a review, see Bjork et al., 2013). In particular, learners frequently rely on processing fluency when evaluating learning effectiveness (Koriat, 1997). Conditions that make learning feel easy often receive high JOLs despite producing relatively poor long-term retention. Conversely, desirable difficulties frequently generate lower JOLs even though they enhance memory. Recent work also suggests that the relationship between metacognitive judgments and foreign-language vocabulary learning is complex. Elsden and Witherby (2026) found that making JOLs produced negative reactivity for noncognate vocabulary, apparently in part because learners subsequently allocated study time differently across items. These findings highlight that metacognitive judgments can both reflect learners’ perceptions of learning and influence how they regulate subsequent study. Nonetheless, whether learners similarly misjudge the benefits of variable practice remains largely unknown. Investigating this issue is important because inaccurate beliefs about effective learning strategies may discourage learners from adopting study methods that would ultimately improve learning outcomes.

The previously discussed three major perspectives can be integrated into a common account of why variable retrieval may influence subsequent retention. Varying the direction of retrieval may require learners to retrieve the same information through different retrieval routes, potentially increasing the number of cues available for subsequent access to that information. From an encoding-variability perspective, these multiple retrieval experiences may produce a more varied set of memory traces or retrieval cues. At the same time, alternating retrieval directions introduces a degree of difficulty relative to repeatedly practicing a single retrieval direction. Consistent with the desirable-difficulties framework, this increased difficulty may reduce immediate retrieval performance while enhancing subsequent retention. Finally, from a cue-utilization perspective, learners’ judgments of learning may rely heavily on cues available during practice, such as retrieval fluency. Consequently, learners may underestimate the eventual benefits of variable retrieval if the condition produces relatively effortful retrieval during acquisition. This integrated account yields the prediction that variable retrieval should benefit delayed retention more than would be expected from immediate practice performance alone and that learners’ judgments will not be sensitive to such a benefit.

## 2. The Present Study

The present research examined the effects of variable retrieval practice on foreign-language vocabulary learning and learners’ metacognitive judgments. Participants learned Lithuanian–English word pairs across multiple study–test cycles in which retrieval occurred consistently in one direction across all cycles (constant retrieval practice) or occurred in both directions (variable retrieval practice). Following practice, participants provided judgments of learning before completing a final retention test after either a short (10 min; Exp 1a) or long (24 h; Exp 1b) delay. This design permitted an evaluation of both the learning consequences of variable retrieval practice and learners’ awareness of those benefits. Based on theories of desirable difficulties and variable practice, it was predicted that conditions involving variable retrieval practice would enhance retention, particularly when the later test was more difficult. At the same time, learners’ judgments of learning were expected to be influenced more by the ease of practice than by the conditions that ultimately produced the best retention. By integrating perspectives from desirable difficulties, variable practice, and metacognition, the present study advances our understanding of how variability affects vocabulary learning and whether learners appreciate its benefits.

## 3. Experiment 1a

### 3.1. Method

#### 3.1.1. Participants

Participants were recruited from the psychology subject pool at an R1 university and received course credit for their participation. A total of 92 participants, all native English speakers, participated in the experiment (*M*_Age_ = 20.14, Female = 81.52%). Current sample sizes were determined prospectively based on available recruitment resources and sample sizes used in prior research using similar paired-associate learning paradigms. The aim was to recruit approximately 20–25 participants per practice condition, which provided a feasible sample size given the resources available for participant recruitment while remaining consistent with prior research using similar paradigms (e.g., Koriat, 1997; Schneider et al., 2002; Soderstrom & Bjork, 2014). This target resulted in 23 participants per condition (*N* = 92) in Experiment 1a.

#### 3.1.2. Materials

The experiment utilized 24 Lithuanian–English word pairs from Grimaldi et al. (2010). All English words were concrete nouns with concreteness ratings between 500–700 (Coltheart, 1981). Furthermore, all the English words were more than three characters in length, and none of the words were hyphenated, compound, or cognates. The 24 Lithuanian–English word pairs were not formally equated on lexical frequency, familiarity, or normative learning difficulty. The same word pairs were used across all four practice conditions, however, so item-level characteristics were held constant across conditions. The Lithuanian words averaged 5.67 letters, whereas the English translations averaged 4.67 letters. Appendix A lists all word pairs used in the current experiments.

#### 3.1.3. Design and Procedure

This experiment was conducted in person and utilized a 4 (Condition) × 2 (Item Type) mixed factorial design, with Condition manipulated between subjects and Item Type manipulated within subjects. There were four practice conditions: EEEE, EEDD, DDEE, or DDDD. The letters denote whether, during a given study–test cycle, the word pairs were studied and tested in the easy (“E”) direction (i.e., recalling the English word given its Lithuanian translation) or the difficult (“D”) direction (i.e., recalling the Lithuanian word given its English translation). In the two constant practice conditions, EEEE and DDDD, pairs were always studied and tested in the same direction across all four study–test cycles (easy direction for EEEE and difficult direction for DDDD). In the variable retrieval practice conditions, EEDD and DDEE, participants were tested in both directions. In the EEDD condition, participants were tested in the easy direction during the first two study–test cycles and the difficult direction during the last two study–test cycles; this schedule was reversed in the DDEE condition. Table 1 summarizes the four practice schedules. In total, 23 participants were assigned sequentially to the four experimental conditions in a fixed cycle, such that Participant 1 was assigned to EEEE, Participant 2 to EEDD, Participant 3 to DDEE, Participant 4 to DDDD, Participant 5 to EEEE, and so forth. This procedure was used to distribute participants evenly across conditions. Thus, condition assignment was systematic rather than based on experimenter judgment or preference.

Before the practice phase began, participants were instructed that they would attempt to learn Lithuanian–English word pairs for an eventual final test that would require recalling a mixture of both English and Lithuanian words. During the practice phase, participants attempted to learn 24 Lithuanian–English word pairs across four study–test cycles on a computer. During each cycle, all 24 word pairs were first presented for study, with each pair (e.g., *namas–house*) displayed individually in the center of the screen for 4 s before automatically advancing to the next pair. Participants did not receive any instructions regarding how to learn the pairs, and no additional information about the words was provided (e.g., pronunciations, example sentences, etc.). Next, participants completed a cued-recall test for all the pairs. Each cue word (i.e., the first word of the pair) was displayed one at a time in the center of the screen for 8 s, and participants were instructed to type the corresponding target word (i.e., the second word of the pair) in a text box. Corrective feedback was not provided on any of the tests. Depending on the practice condition, items were tested in either the easy retrieval direction (Lithuanian cue–English target; e.g., *namas–______*), the difficult retrieval direction (English cue–Lithuanian target; e.g., *house–______*), or a combination of both directions across the four study–test cycles. The order of the word pairs was randomized separately for each participant during every study and test phase.

After completing the fourth study–test cycle, participants provided a judgment of learning (JOL) by predicting how many of the 24 word pairs they thought they would remember on a forthcoming final test. The exact question read, “In 10 min, you will have the final test. How many of the 24 word pairs do you think you will remember at that time?” Participants typed their JOL in a text box under the question and then pressed “enter” on the keyboard to submit their response. Next, participants played a 10 min game of Tetris, which served as a distractor task.

Following the 10 min retention interval, participants completed a final cued-recall test. All 24 word pairs were tested, with half of the items presented in the easy retrieval direction and half presented in the difficult retrieval direction. Each cue word was displayed individually and remained on the screen for 8 s, during which time participants typed the target word corresponding to the cue. Assignment of items to retrieval direction and the order of test presentation were randomized for each participant. After the final test, participants were debriefed and thanked for their participation.

### 3.2. Results

A relatively lenient automatic scoring system was used such that minor spelling mistakes and typographical errors were counted as correct. More specifically, the program allowed for one deletion, addition, substitution, or transposition. Letter capitalization did not matter. Furthermore, response times indicated that the 8 s response window for recall was not a limiting factor. Across both experiments, mean response times did not exceed 3.5 s for either English or Lithuanian targets across practice blocks and on the final test.

#### 3.2.1. Practice Performance

**Figure 1:**
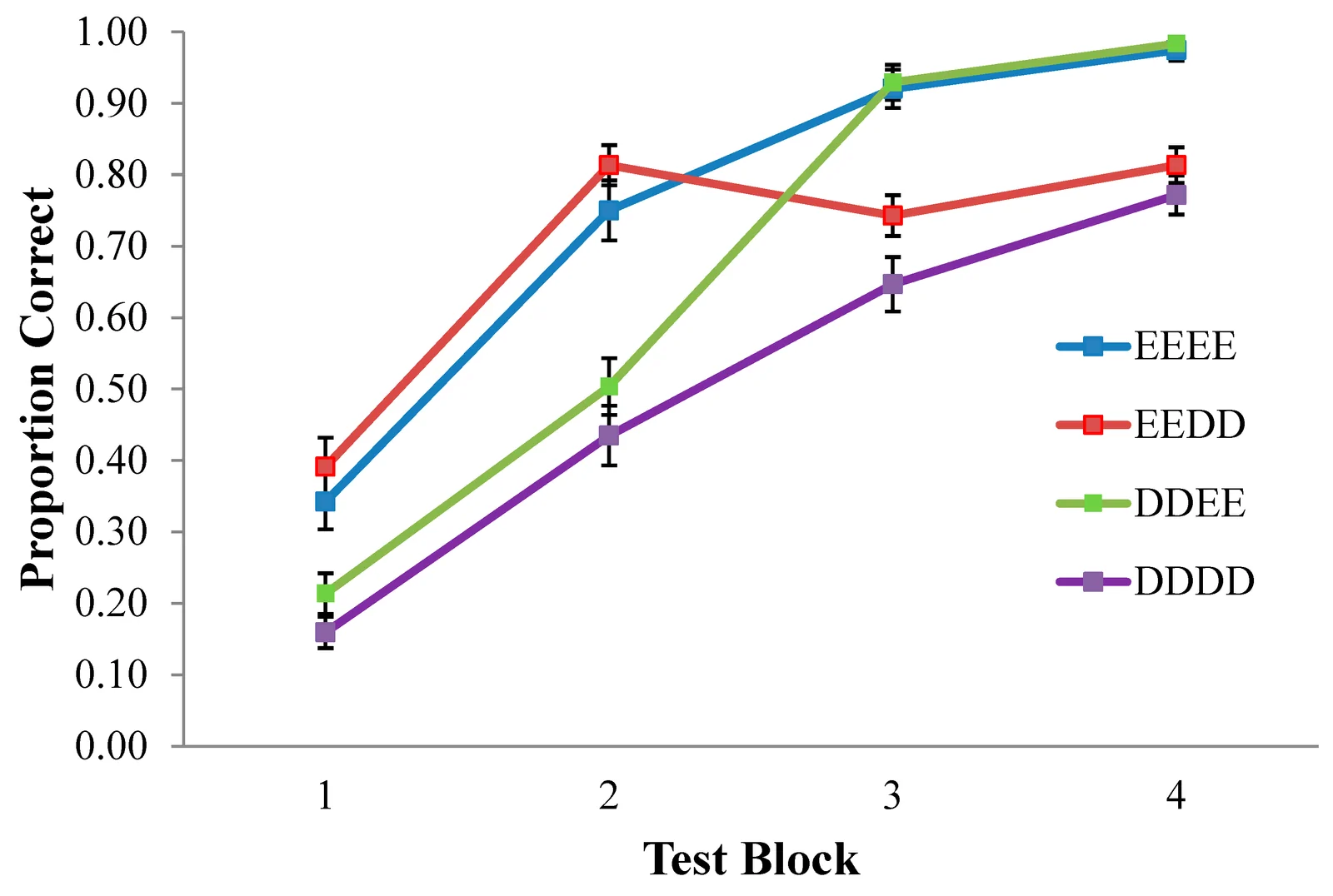
Mean proportion of items correctly recalled across the four testing blocks during the practice phase in Experiment 1a. *Note*. EEEE = tested in the easy direction for all four testing blocks; EEDD = tested in the easy direction for the first two testing blocks and in the difficult direction for the last two testing blocks; DDEE = tested in the difficult direction for the first two testing blocks and in the easy direction for the last two testing blocks; DDDD = tested in the difficult direction for all four testing blocks. Error bars denote standard error of the mean.

Figure 1 presents participants’ mean correct recall across the four test blocks during the practice phase. A 4 (Condition) × 4 (Test Block) mixed-factor ANOVA revealed a main effect of Condition, *F*(3, 88) = 15.93, *p* < 0.001, η_p_^2^ = 0.35, and a main effect of Test Block, *F*(3, 264) = 774.12, *p* < 0.001, η_p_^2^ = 0.90. However, these main effects were qualified by a significant interaction, *F*(9, 264) = 29.16, *p* < 0.001, η_p_^2^ = 0.50.

Follow-up one-way ANOVAs revealed an effect of Condition in test block 1, *F*(3, 88) = 10.64, *p* < 0.001, η_p_^2^ = 0.27; in test block 2, *F*(3, 88) = 23.26, *p* < 0.001, η_p_^2^ = 0.44; in test block 3, *F*(3, 88) = 21.57, *p* < 0.001, η_p_^2^ = 0.42; and in test block 4, *F*(3, 88) = 28.35, *p* < 0.001, η_p_^2^ = 0.49. Tukey’s HSD post hoc comparisons revealed that in test block 1, both the EEEE and EEDD conditions recalled more items than the DDEE (*p* = 0.04, *d* = 0.79, 95% CI [0.19, 1.39]; *p* = 0.002, *d* = 1.05, 95% CI [0.43, 1.67], respectively) and DDDD (*p* = 0.001, *d* = 1.22, 95% CI [0.58, 1.84]; *p* < 0.001, *d* = 1.48, 95% CI [0.82, 2.13], respectively) conditions. A similar pattern persisted in test block 2, with the EEEE condition outperforming the DDEE and DDDD conditions (*p* < 0.001, *d* = 1.26, 95% CI [0.62, 1.89]; *p* < 0.001, *d* = 1.57, 95% CI [0.90, 2.23], respectively) and the EEDD condition outperforming the DDEE and DDDD conditions (*p* < 0.001, *d* = 1.88, 95% CI [1.17, 2.57]; *p* < 0.001, *d* = 2.22, 95% CI [1.47, 2.95], respectively). In test block 3, the EEEE condition recalled more items than the EEDD (*p* < 0.001, *d* = 1.34, 95% CI [0.69, 1.97]) and DDDD (*p* < 0.001, *d* = 1.74, 95% CI [1.05, 2.41] conditions, and the DDEE condition also outperformed the EEDD (*p* < 0.001, *d* = 1.46, 95% CI [0.80, 2.10]) and DDDD (*p* < 0.001, *d* = 1.84, 95% CI [1.14, 2.53] conditions. The same pattern was observed in test block 4: the EEEE condition outperformed the EEDD (*p* < 0.001; *d* = 1.63, 95% CI [0.96, 2.30] and DDDD (*p* < 0.001; *d* = 1.92, 95% CI [1.21, 2.62] conditions, and the DDEE condition outperformed the EEDD (*p* < 0.001; *d* = 1.91, 95% CI [1.20, 2.61] and DDDD (*p* < 0.001; *d* = 2.19, 95% CI [1.45, 2.92] conditions.

Follow-up one-way ANOVAs were also performed to examine how acquisition performance changed across test blocks for each condition. A significant effect of Test Block was observed for all the conditions. In both the EEEE condition, *F*(3, 66) = 165.22, *p* < 0.001, η_p_^2^ = 0.88, and DDDD condition, *F*(3, 66) = 231.27, *p* < 0.001, η_p_^2^ = 0.91, post hoc analyses revealed that recall increased across each subsequent test block (all *p*s < 0.001; EEEE: *d*_z_ = 2.78, 95% CI [1.86, 3.69], 1.43, 95% CI [0.84, 2.01], and 0.56, 95% CI [0.11, 0.99] for blocks 1–2, 2–3, and 3–4, respectively; DDDD: *d*_z_ = 2.00, 95% CI [1.28, 2.71], 2.41, 95% CI [1.59, 3.22], and 1.31, 95% CI [0.74, 1.86] for blocks 1–2, 2–3, and 3–4, respectively). In the EEDD condition, *F*(3, 66) = 121.40, *p* < 0.001, η_p_^2^ = 0.85, recall increased from block 1 to block 2 (*p* < 0.001, *d*_z_ = 3.44, 95% CI [2.35, 4.53]), declined from block 2 to block 3 (*p* = 0.02, *d*_z_ = −0.71, 95% CI [−1.16, −0.25]), and then increased again from block 3 to block 4 (*p* < 0.001, *d*_z_ = 1.04, 95% CI [0.52, 1.54]). The drop in performance from block 2 to block 3 was likely due to the fact that block 3 was the first time these participants attempted to recall the pairs in the difficult direction. Finally, in the DDEE condition, *F*(3, 66) = 350.19, *p* < 0.001, η_p_^2^ = 0.94, recall increased across the first three test blocks (*p*s < 0.001; *d*_z_ = 2.40, 95% CI [1.58, 3.21] and *d*_z_ = 3.02, 95% CI [2.04, 3.99] for blocks 1–2 and 2–3, respectively) and then leveled off after test block 3 (*p* = 0.054, *d*_z_ = 0.60, 95% CI [0.15, 1.04]).

To summarize the practice phase results, recall improved significantly across all four conditions; however, performance varied as a function of retrieval difficulty. Participants tested in the easy retrieval direction during a given block consistently outperformed those tested in the difficult direction. As expected, the EEEE and EEDD groups showed superior recall during the first two test blocks, whereas the EEEE and DDEE groups performed best during the final two blocks. By the end of practice, recall in the EEEE and DDEE conditions approached 100%.

#### 3.2.2. Final Test Performance

**Figure 2:**
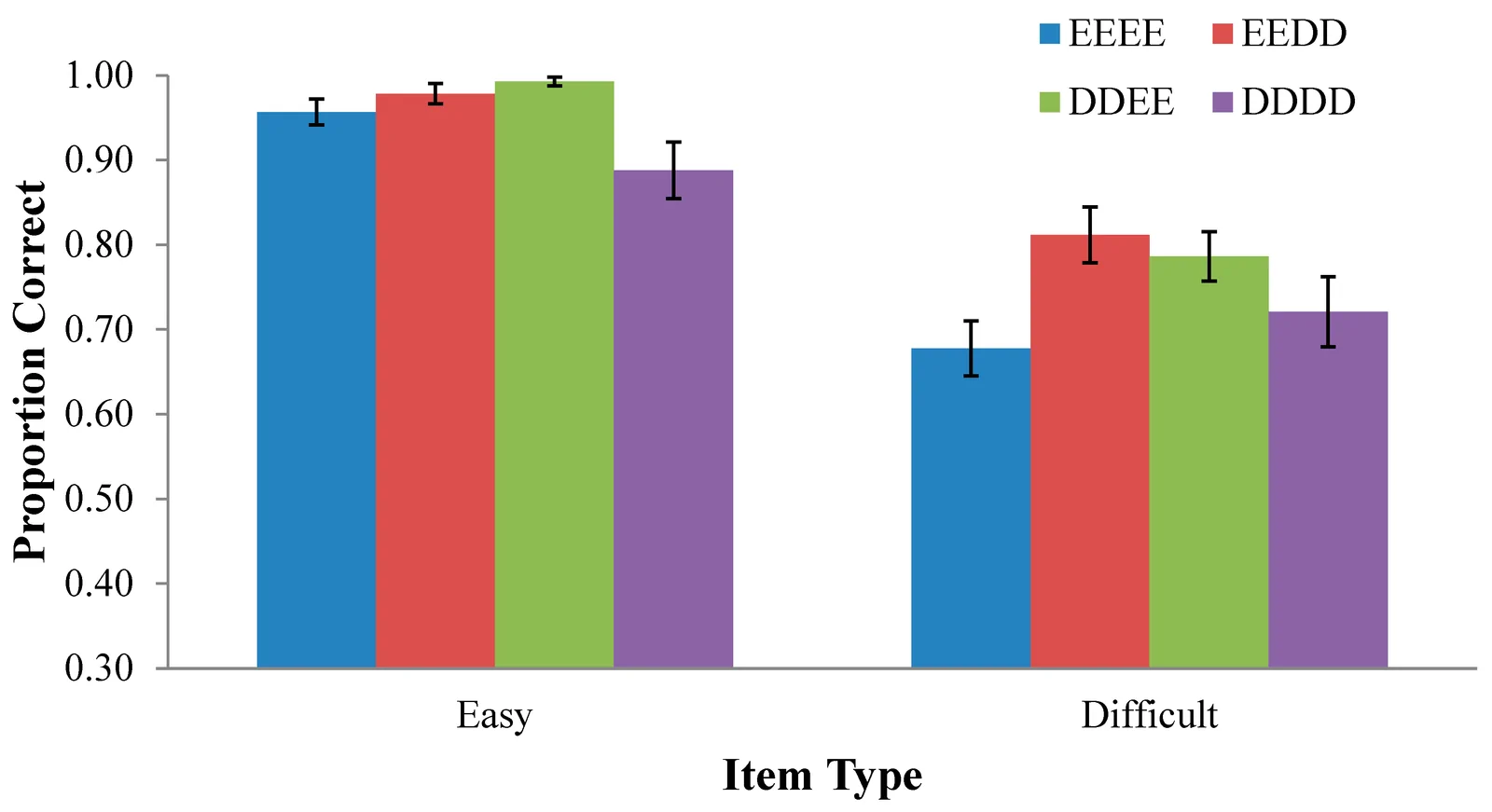
Mean proportion of items correctly recalled on the final cued-recall test in Experiment 1a. *Note*. EEEE = tested in the easy direction for all four testing blocks; EEDD = tested in the easy direction for the first two testing blocks and in the difficult direction for the last two testing blocks; DDEE = tested in the difficult direction for the first two testing blocks and in the easy direction for the last two testing blocks; DDDD = tested in the difficult direction for all four testing blocks. Error bars denote standard error of the mean.

Figure 2 shows final test performance across each condition for items tested in the easy direction (i.e., recalling the English word given the Lithuanian word) and difficult direction (i.e., recalling the Lithuanian word given the English word). A 4 (Condition) × 2 (Item Type) mixed-factor ANOVA revealed a main effect of Condition, *F*(3, 88) = 4.08, *p* = 0.009, η_p_^2^ = 0.12, and a main effect of Item Type, *F*(1, 88) = 190.74, *p* < 0.001, η_p_^2^ = 0.68. However, these main effects were qualified by a significant interaction, *F*(3, 88) = 3.23, *p* = 0.03, η_p_^2^ = 0.10.

To examine the interaction, separate one-way ANOVAs were performed for the easy and difficult items. For the easy items, Condition significantly affected recall, *F*(3, 88) = 5.73, *p* < 0.001, η_p_^2^ = 0.16; Tukey’s HSD comparisons showed that the EEEE, EEDD, and DDEE conditions all recalled more items than the DDDD condition (*p* = 0.03, *d* = 0.55, 95% CI [−0.04, 1.14]; *p* = 0.008, *d* = 0.75, 95% CI [0.15, 1.35]; and *p* = 0.001, *d* = 0.92, 95% CI [0.30, 1.52], respectively). A planned comparison examining the potential difference between the two groups that finished practice with easy retrieval showed that participants in the DDEE condition recalled more easy items than participants in the EEEE condition, *t*(44) = 2.25, *p* = 0.03, *d* = 0.66, 95% CI [0.07, 1.25], indicating that variable retrieval practice may benefit easy later recall.

There was also an effect of Condition for the difficult items, *F*(3, 88) = 3.24, *p* = 0.03, η_p_^2^ = 0.10. Post hoc analyses revealed that the EEDD condition recalled more items than the EEEE condition (*p* = 0.03, *d* = 0.86, 95% CI [0.25, 1.46]). A planned comparison revealed that the EEDD condition did not differ from the DDDD condition, *t*(44) = 1.73, *p* = 0.09, *d* = 0.51, 95% CI [−0.08, 1.10]. Finally, there was no difference between the EEDD and DDEE conditions, *t*(44) = 0.58, *p* = 0.56, *d* = 0.17, 95% CI [−0.41, 0.75].

To summarize the final test results in Experiment 1a, variable retrieval practice conferred some benefits over constant retrieval practice, although it did not produce a consistent advantage. For easy items, performance was higher following DDEE than EEEE practice. For difficult items, however, performance did not differ between EEDD and DDDD. Thus, although Experiment 1a showed evidence that variable retrieval practice may benefit easy items after a short retention interval, there was no evidence that either variable-practice condition produced better performance for difficult items.

#### 3.2.3. JOLs

Table 2 shows the JOL data. To test whether different JOLs were given across the four conditions, a one-way ANOVA was performed, which revealed an effect of Condition, *F*(3, 88) = 3.75, *p* = 0.01, η_p_^2^ = 0.11. The only significant difference was that participants in the EEEE condition provided higher JOLs than those in the DDDD condition, *t*(44) = 3.21, *p* = 0.002, *d* = 0.95, 95% CI [0.33, 1.55]. Additionally, there was a significant correlation between mean practice performance and JOLs, *r*(90) = 0.67, *p* < 0.001. These data suggest that participants’ predictions may have been influenced by the perceived ease of practice. Participants’ JOLs were also correlated with overall final test performance, *r*(90) = 0.47, *p* < 0.001, suggesting that JOLs were relatively predictive of subsequent final recall.

## 4. Experiment 1b

Experiment 1a provided some evidence that variable retrieval may benefit later recall. When the later test was easy, the DDEE condition recalled more items than the EEEE condition. When the later test was difficult, the results were somewhat unclear because while the EEDD condition recalled more difficult items than the EEEE condition, the EEDD condition did not outperform the DDDD condition. Additionally, JOLs seemed to have reflected ease of practice performance and were correlated with subsequent final recall. Experiment 1b employed the same study design as Experiment 1a but extended the retention interval to 24 h to test whether, as suggested by the desirable-difficulties framework, the learning benefits of variable practice emerge after a delay, especially when the retention test is relatively challenging. Two follow-up metacognitive questions were also added to the end of Experiment 1b.

### 4.1. Method

#### 4.1.1. Participants

Participants were recruited from the psychology subject pool at an R1 university and received course credit for their participation. As in Experiment 1a, the sample size was determined prospectively based on available recruitment resources and prior research using similar paired-associate learning paradigms. The aim was again to recruit approximately 20–25 participants per practice condition, resulting in 22 participants per condition (*N* = 88). All participants were native English speakers (*M*_Age_ = 20.42, Female = 79.54%).

#### 4.1.2. Materials

The same word pairs used in Experiment 1a were also used in Experiment 1b (see Appendix A).

#### 4.1.3. Design and Procedure

Experiment 1b was identical to Experiment 1a, with three exceptions. First, the retention interval between the practice phase and final test was 24 h, rather than 10 min. Although additional study materials or instructions for reviewing the vocabulary pairs were not provided to participants during the intervening period, no formal controls or post-experiment screening procedures were implemented to monitor participants’ activities during this interval. Second, the JOL prompt was modified to reflect the new retention interval (“In 24 h, you will have the final test. How many of the 24 word pairs do you think you will remember at that time?”). Finally, immediately after the final test, participants received descriptions of the four practice conditions used in the current experiment and were then asked to respond to the following two metacognitive questions at their own pace:

1. “Which strategy would be most helpful for this task?”
2. “When in this type of situation (i.e., when you need to learn vocabulary words), which schedule of learning is most similar to what you personally use?”

### 4.2. Results

#### 4.2.1. Practice Performance

**Figure 3:**
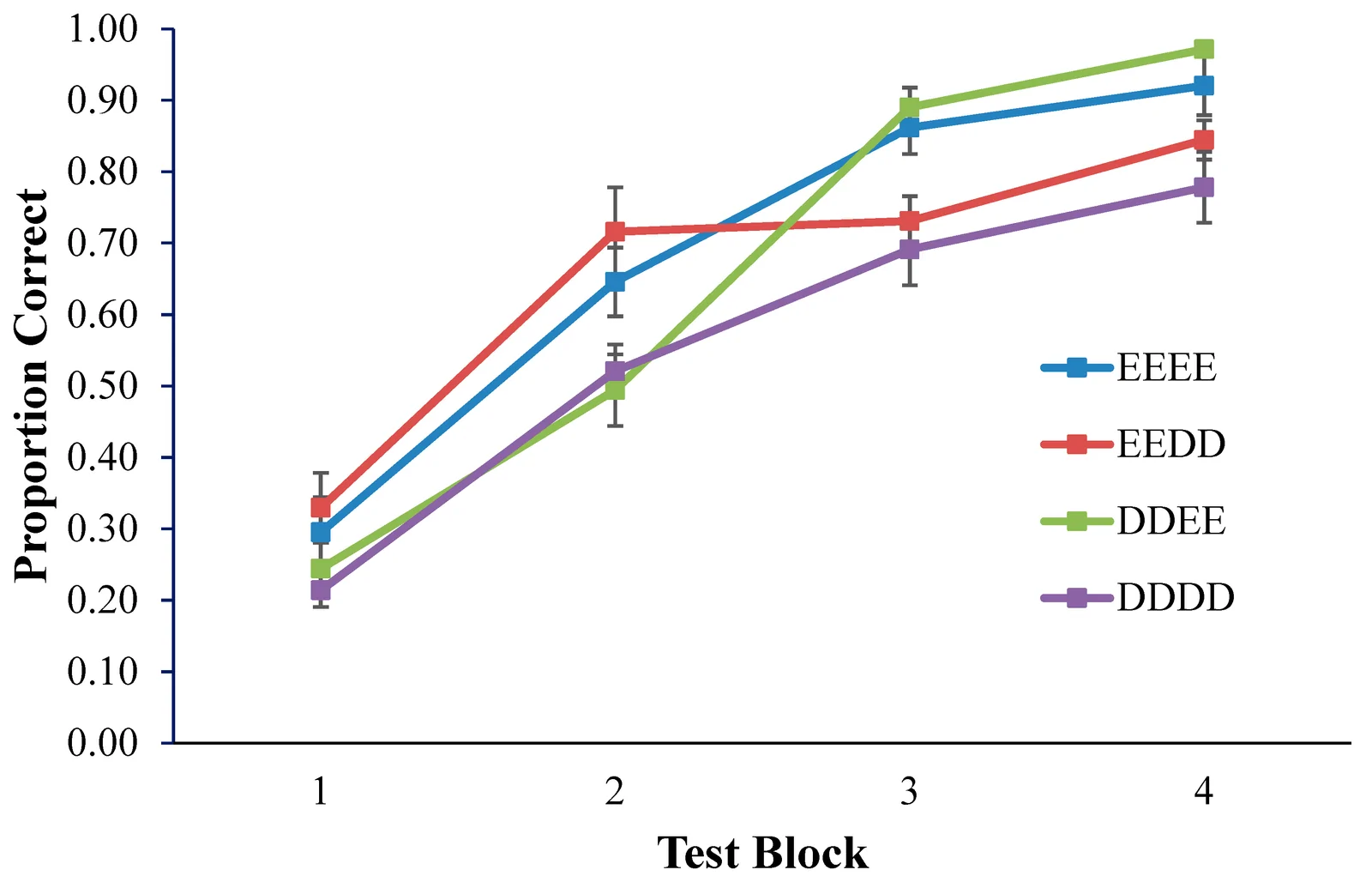
Mean proportion of items correctly recalled across the four testing blocks during the practice phase in Experiment 1b. *Note*. EEEE = tested in the easy direction for all four testing blocks; EEDD = tested in the easy direction for the first two testing blocks and in the difficult direction for the last two testing blocks; DDEE = tested in the difficult direction for the first two testing blocks and in the easy direction for the last two testing blocks; DDDD = tested in the difficult direction for all four testing blocks. Error bars denote standard error of the mean.

Figure 3 presents participants’ mean correct recall across the four test blocks during the practice phase. A 4 (Condition) × 4 (Test Block) mixed-factor ANOVA revealed no main effect of Condition, *F*(3, 84) = 2.45, *p* = 0.07, η_p_^2^ = 0.08, but the main effect of Test Block was reliable, *F*(3, 252) = 519.44, *p* < 0.001, η_p_^2^ = 0.86. The interaction was also significant, *F*(9, 252) = 10.02, *p* < 0.001, η_p_^2^ = 0.26.

Follow-up one-way ANOVAs were conducted to examine the effects of the learning conditions in each test block. There was no significant effect of Condition in test block 1, *F*(3, 84) = 1.39, *p* = 0.25, η_p_^2^ = 0.05, indicating that all of the conditions started at similar levels of recall. There were, however, significant effects of Condition in test block 2, *F*(3, 84) = 4.30, *p* = 0.01, η_p_^2^ = 0.13; in test block 3, *F*(3, 84) = 6.50, *p* < 0.001, η_p_^2^ = 0.18; and in test block 4, *F*(3, 84) = 5.75, *p* < 0.001, η_p_^2^ = 0.17. Tukey’s HSD post hoc comparisons revealed that in test block 2, the EEDD condition recalled more items than the DDEE (*p* = 0.01, *d* = 0.84, 95% CI [0.21, 1.45]) and DDDD (*p* = 0.04, *d* = 0.81, 95% CI [0.19, 1.42]) conditions. In test block 3, the DDEE condition recalled more items than the EEDD (*p* = 0.02, *d* = 1.08, 95% CI [0.44, 1.70]) and DDDD (*p* = 0.002, *d* = 1.05, 95% CI [0.41, 1.67]) conditions. Finally, in test block 4, both the EEEE and the DDEE conditions recalled more items than the DDDD condition (*p* = 0.03, *d* = 0.67, 95% CI [0.05, 1.27]; *p* = 0.001, *d* = 1.16, 95% CI [0.51, 1.79], respectively).

Follow-up one-way ANOVAs were also performed to examine how acquisition performance changed across test blocks for each condition. A significant effect of Test Block was observed for all the conditions. In the EEEE condition, *F*(3, 63) = 120.01, *p* < 0.001, η_p_^2^ = 0.85, the DDEE condition, *F*(3, 63) = 190.76, *p* < 0.001, η_p_^2^ = 0.90, and the DDDD condition, *F*(3, 63) = 144.45, *p* < 0.001, η_p_^2^ = 0.87, post hoc analyses revealed that recall increased across each subsequent test block (all *p*s < 0.001; EEEE: *d*_z_ = 2.91, 95% CI [1.93, 3.87], 1.48, 95% CI [0.86, 2.08], and 0.88, 95% CI [0.38, 1.37] for blocks 1–2, 2–3, and 3–4, respectively; DDEE: *d*_z_ = 2.55, 95% CI [1.67, 3.42], 2.36, 95% CI [1.53, 3.18], and 0.77, 95% CI [0.28, 1.24] for blocks 1–2, 2–3, and 3–4, respectively; DDDD: *d*z = 2.82, 95% CI [1.87, 3.76], 1.61, 95% CI [0.96, 2.24], and 1.03, 95% CI [0.51, 1.55] for blocks 1–2, 2–3, and 3–4, respectively). In the EEDD condition, *F*(3, 63) = 93.95, *p* < 0.001, η_p_^2^ = 0.82, recall increased from block 1 to block 2 (*p* < 0.001, *d*_z_ = 2.66, 95% CI [1.75, 3.55]); remained the same from block 2 to block 3 (*p* = 1.00, *d*_z_ = 0.10, 95% CI [−0.32, 0.52]); and then increased again from block 3 to block 4 (*p* < 0.001, *d*_z_ = 1.36, 95% CI [0.77, 1.94]).

Taken together, the acquisition results from Experiment 1b are generally consistent with those from Experiment 1a. Recall improved significantly across all four conditions, but performance varied as a function of retrieval difficulty, with participants who ended practice with easy retrieval (EEEE and DDEE) outperforming those who ended with difficult retrieval practice (EEDD and DDDD).

#### 4.2.2. Final Test Performance

**Figure 4:**
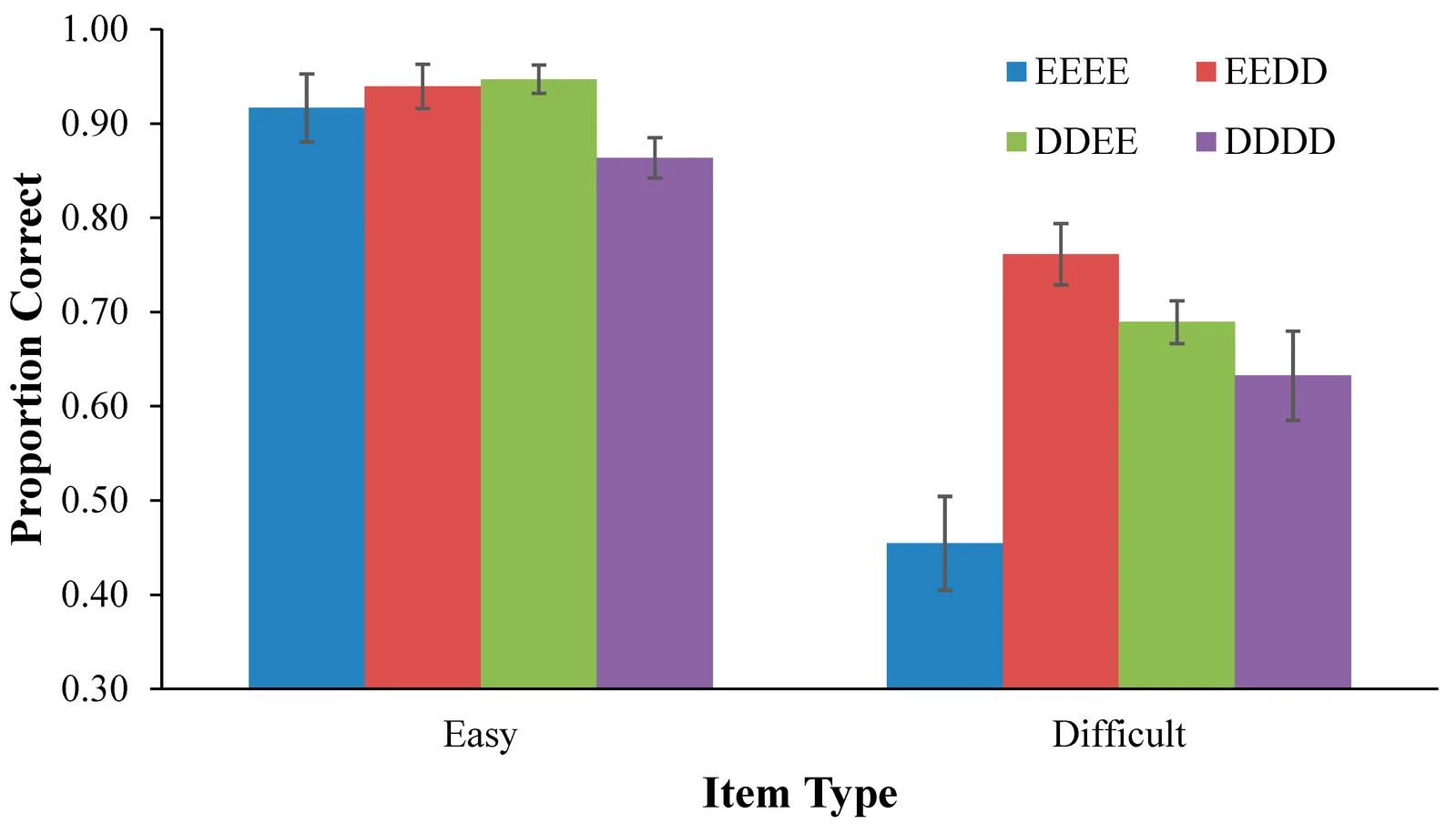
Mean proportion of items correctly recalled on the final cued-recall test in Experiment 1b. *Note*. EEEE = tested in the easy direction for all four testing blocks; EEDD = tested in the easy direction for the first two testing blocks and in the difficult direction for the last two testing blocks; DDEE = tested in the difficult direction for the first two testing blocks and in the easy direction for the last two testing blocks; DDDD = tested in the difficult direction for all four testing blocks. Error bars denote standard error of the mean.

Figure 4 shows final test performance for each practice condition for items tested in the easy and difficult retrieval directions. A 4 (Condition) × 2 (Item Type) mixed-factor ANOVA revealed a main effect of Condition, *F*(3, 84) = 6.95, *p* < 0.001, η_p_^2^ = 0.20, as well as a main effect of Item Type, *F*(1, 84) = 247.38, *p* < 0.001, η_p_^2^ = 0.75. However, these main effects were qualified by a significant interaction, *F*(3, 84) = 12.08, *p* < 0.001, η_p_^2^ = 0.30.

To examine the interaction, separate one-way ANOVAs were performed for the easy and difficult items. For the easy items, there was no effect of Condition, *F*(3, 84) = 2.32, *p* = 0.08, η_p_^2^ = 0.08, and the planned comparison between the DDEE and EEEE conditions showed no difference, *t*(42) = 0.77, *p* = 0.44, *d* = 0.23, 95% CI [−0.36, 0.83], indicating that variable retrieval practice did not benefit easy retrieval, although this result should be interpreted with caution as ceiling effects may have prevented the detection of a difference. However, there was a significant effect of Condition for the difficult items, *F*(3, 84) = 10.90, *p* < 0.001, η_p_^2^ = 0.28. Post hoc analyses revealed that the EEDD, DDEE, and DDDD conditions recalled more items than the EEEE condition (*p* < 0.001, *d* = 1.55, 95% CI [0.87, 2.22]; *p* < 0.001, *d* = 1.29, 95% CI [0.63, 1.94]; and *p* = 0.01, *d* = 0.78, 95% CI [0.16, 1.39], respectively). A planned comparison examining the potential difference between the two groups that finished practice with difficult retrieval showed that participants in the EEDD condition recalled more difficult items than participants in the DDDD condition, *t*(42) = 2.25, *p* = 0.03, *d* = 0.68, 95% CI [0.07, 1.28], indicating that variable retrieval practice benefited later difficult recall. Like Experiment 1a, there was no difference between the EEDD and DDEE conditions, *t*(42) = 1.80, *p* = 0.08, *d* = 0.54, 95% CI [−0.06, 1.14]. Thus, despite producing relatively low acquisition performance, variable retrieval practice enhanced vocabulary learning after a 24 h retention interval when the later test was challenging.

#### 4.2.3. JOLs and Follow-Up Metacognitive Questions

Table 2 shows the JOL data for Experiment 1b. To test whether different JOLs were given across the four practice conditions, a one-way ANOVA was performed, and it revealed no effect of Condition, *F*(3, 84) = 0.94, *p* = 0.43, η_p_^2^ = 0.03. Thus, unlike the result from Experiment 1a, in which participants in the EEEE condition gave higher JOLs than those in the DDDD condition, participants gave equivalent JOLs across all four practice conditions in Experiment 1b. Furthermore, as in Experiment 1a, mean practice performance correlated with JOLs, *r*(86) = 0.23, *p* = 0.03. However, unlike in Experiment 1a, there was no significant correlation between JOLs and final test performance, *r*(86) = 0.08, *p* = 0.46.

Table 3 presents the responses to the two follow-up metacognitive questions. As is evident, participants endorsed the two variable practice conditions as being more helpful with the current task than the two constant practice conditions. The easy-to-difficult variable condition accumulated the most support, whereas the exclusively difficult constant condition garnered the least support. Turning to the question about which strategy participants use on their own when studying vocabulary words, nearly 40% indicated that they use a strategy that resembles the easy-to-difficult variable condition. The next most popular strategy was the exclusively easy strategy, followed by the exclusively difficult strategy. Few participants reported using the difficult-to-easy strategy on their own. Taken together, these results may suggest that learners recognize the effectiveness of variable retrieval practice and that many learners are already using forms of variable retrieval practice on their own. However, it may also be the case that these judgments simply reflect a retrospective belief or preference, not necessarily awareness of the best strategy.

## 5. General Discussion

The present research examined whether varying retrieval direction during foreign-language vocabulary learning improves retention and, if it does, whether learners appreciate the benefits of such variability. Across two experiments, participants learned Lithuanian–English word pairs using either constant retrieval practice (always retrieving in the easy or difficult direction) or variable retrieval practice (retrieving in both directions across study–test cycles). Several consistent patterns emerged. First, practice performance was strongly influenced by retrieval difficulty. Participants consistently recalled more items when practice occurred in the easier retrieval direction than in the more difficult direction, replicating Schneider et al. (2002) results, and groups that ended practice with easy retrieval demonstrated the highest levels of acquisition performance.

Importantly, superior practice performance did not translate into superior delayed retention, illustrating the distinction between learning and performance emphasized in the desirable-difficulties framework (Bjork, 1994; Soderstrom & Bjork, 2015). Despite producing relatively lower levels of recall during portions of the practice phase, incorporating retrieval difficulty was shown to enhance recall on the final test. Furthermore, although both current experiments supplied evidence for the benefit of variable retrieval practice, the pattern of results differed descriptively across retention intervals, with the clearest evidence emerging following the 24 h retention interval (Exp 1b). Importantly, the two variable-practice conditions did not differ from one another in either experiment, suggesting that experiencing retrieval in both directions was more important than the order in which the retrieval directions were practiced. Taken together, these findings suggest that variable retrieval practice may facilitate retention of foreign-language vocabulary, especially when learners are required to remember information after a delay and under challenging conditions.

The current results extend previous work on desirable difficulties and variable practice to foreign-language vocabulary learning (Bjork & Kroll, 2015). Whereas Schneider et al. (2002) demonstrated benefits of consistently retrieving vocabulary in a difficult direction, the present findings indicate that varying retrieval difficulty during practice can also improve retention. This pattern is consistent with theoretical accounts proposing that more diverse encoding conditions promoted by variable practice provide a broader range of retrieval cues (Estes, 1955; Raviv et al., 2022). Likewise, varying retrieval direction may encourage learners to form more flexible bidirectional associations between foreign-language vocabulary pairs. Rather than repeatedly practicing a single retrieval pathway, learners appear to benefit from experiencing multiple routes to the same information.

An alternative interpretation of the present findings is that the observed benefits reflect exposure to the more difficult retrieval direction rather than retrieval variability per se. Indeed, both variable retrieval practice conditions required participants to retrieve the Lithuanian target during portions of practice, and such experience may have contributed to better performance on difficult final-test items through greater practice-test congruency. However, in Experiment 1b, participants in the easy-to-difficult (EEDD) variable retrieval condition outperformed those who practiced exclusively in the difficult direction (DDDD), suggesting that difficult-direction exposure alone does not fully account for the observed pattern. Nevertheless, the relative contributions of retrieval variability, retrieval difficulty, and practice-test congruency remain unsettled empirical questions. Future research should experimentally dissociate these factors to determine the conditions under which each contributes most strongly to long-term retention.

The metacognitive findings further highlight an important distinction between subjective learning experiences and objective learning outcomes. Participants’ JOLs did not appear to anticipate the relative advantages of variable retrieval practice. In Experiment 1a, learners who practiced exclusively in the easy direction expressed greater confidence than those who practiced exclusively in the difficult direction, a pattern that did not materialize on the final test. Although this difference was not replicated in Experiment 1b, JOLs did not differentiate among the learning conditions in a manner that corresponded to their final-test performance, as evidenced by equivalent JOLs across all four practice conditions. Furthermore, there was correlational evidence across both experiments that participants used practice performance to inform their JOLs. These findings are consistent with the cue-utilization account of JOLs (Koriat, 1997) and prior research demonstrating that learners frequently rely on the subjective ease or fluency of practice when evaluating their own learning (for a review, see Bjork et al., 2013). The present findings also add to recent evidence that metacognitive judgments do not necessarily correspond with the conditions that optimize foreign-language vocabulary learning. Whereas Elsden and Witherby (2026) showed that making JOLs can alter subsequent study behavior in ways that are not uniformly beneficial, the present findings suggest that learners may also fail to anticipate the relative effectiveness of different retrieval-practice schedules. Alternatively, the current JOL findings may have resulted, at least in part, because the practice conditions were manipulated between subjects, which meant that participants could not make relative comparisons between their assigned practice condition and any of the other conditions (Koriat et al., 2004). Future research might test this possibility by manipulating the current practice conditions within subjects.

Interestingly, participants’ beliefs appeared to become more accurate once they had completed the experiment and were explicitly asked to evaluate the different practice schedules. Nearly 40% of participants identified the easy-to-difficult variable schedule as the most effective practice strategy in the current context, and many reported already using a similar approach when studying vocabulary on their own. These responses suggest that some learners may possess an appreciation for the value of variable practice once they are encouraged to reflect on alternative learning schedules. Thus, although prospective judgments made during (or shortly after) acquisition may rely heavily on immediate performance cues, retrospective evaluations may better capture learners’ beliefs about effective study strategies. This would be consistent with Koriat’s (1997) distinction between experience- and theory-based judgments. Future work should investigate whether providing learners with experience or feedback about desirable difficulties improves their ability to select effective learning strategies or if such judgments simply reflect retrospective beliefs or preferences.

The current study has several potential limitations that should be acknowledged. First, the experiments examined a relatively narrow form of variability, which involved alternating retrieval direction while learning paired-associate vocabulary. Whether similar benefits generalize to other educational materials, such as prose passages, conceptual knowledge, or procedural skills, remains to be determined. Second, the longest retention interval examined in the current study was 24 h, and the final test used the same word pairs that participants encountered during practice. Thus, the study demonstrates retention of the practiced vocabulary and transfer across retrieval direction, but it does not establish transfer to novel vocabulary or new learning tasks. Future research using longer retention intervals and novel materials will be needed to determine the durability of retrieval variability effects and whether their benefits generalize more broadly. Third, the present study was not designed to distinguish among potential mechanisms underlying the effects of variable retrieval practice. Future research should examine whether retrieval variability enhances learning by strengthening bidirectional associations, increasing retrieval cue diversity, encouraging elaborative processing, or some combination of these mechanisms. Fourth, near-ceiling level performance on the easy test may have limited the ability to detect condition differences; thus, the results corresponding to the easy test should be interpreted with caution. Finally, a limitation of Experiment 1b, specifically, is that participants’ activities during the 24 h retention interval could not be controlled. Although participants were not provided with the vocabulary materials for additional study, incidental exposure to the materials during the delay period cannot be ruled out.

From an applied perspective, the present findings suggest that vocabulary instruction may benefit from incorporating variable retrieval practice rather than relying exclusively on a single retrieval format. Language-learning applications, flashcard systems, and classroom activities often require learners to retrieve information in only one direction, even though real-world language use requires flexible access to both the native and foreign language. Incorporating retrieval in both directions may not only promote more durable retention, but it may also better prepare learners to use vocabulary flexibly across communicative contexts, although much more research is needed to test these possibilities. More broadly, these findings reinforce the principle that practice conditions that appear less successful during acquisition may ultimately produce better learning.

## 6. Conclusions

Across two experiments, varying retrieval direction during vocabulary acquisition enhanced retention despite producing lower performance during acquisition. The pattern of results differed descriptively across retention intervals, with the clearest evidence for benefits of variable retrieval practice emerging following a 24 h retention interval. These findings extend the literature on desirable difficulties and variable practice by demonstrating that retrieval variability benefits foreign-language vocabulary learning, particularly when the later test is delayed and challenging. At the same time, learners’ prospective metacognitive judgments generally failed to anticipate these benefits, highlighting a persistent disconnect between subjective learning experiences and objective learning outcomes. Together, the results suggest the intriguing possibility that introducing variability into retrieval practice represents a simple yet effective way to promote retention while underscoring the importance of helping learners recognize that conditions making learning feel more difficult can sometimes produce greater learning benefits.

## Figures and Tables

**Table 1:** The four practice conditions used in Experiments 1a and 1b. Conditions EEEE and DDDD represent constant retrieval practice conditions; conditions EEDD and DDEE represent variable retrieval practice conditions.

	Study–Test Cycle			
Practice Condition	1	2	3	4
EEEE	Easy	Easy	Easy	Easy
EEDD	Easy	Easy	Difficult	Difficult
DDEE	Difficult	Difficult	Easy	Easy
DDDD	Difficult	Difficult	Difficult	Difficult

*Note.* “E” = easy direction (i.e., recalling the English word given its Lithuanian translation; “D” = difficult direction (i.e., recalling the Lithuanian word given its English translation).

**Table 2:** Mean judgments of learning (JOLs) in Experiments 1a and 1b.

	Experiment 1a	Experiment 1b
EEEE	19.09 (5.13)	15.45 (7.02)
EEDD	16.57 (4.58)	12.64 (5.94)
DDEE	15.70 (5.62)	13.55 (4.39)
DDDD	14.00 (5.60)	14.18 (5.30)

*Note*. EEEE = tested in the easy direction for all four testing blocks; EEDD = tested in the easy direction for the first two testing blocks and in the difficult direction for the last two testing blocks; DDEE = tested in the difficult direction for the first two testing blocks and in the easy direction for the last two testing blocks; DDDD = tested in the difficult direction for all four testing blocks. Standard deviations are reported in parentheses.

**Table 3:** Responses to follow-up metacognitive questions in Experiment 1b.

Question	EEEE	EEDD	DDEE	DDDD
Strategy Most Helpful With This Task?	20.45%	39.77%	32.95%	6.82%
Strategy Most Like What You Use?	26.14%	38.64%	14.77%	20.45%

*Note*. All four practice conditions were explained to participants before the questions were answered. Abbreviated versions of the questions are reported in the table for brevity; the full questions can be found in the Section 4.1 of Experiment 1b.

## Data Availability

The raw data supporting the conclusions of this article will be made available by the authors on request.

## Appendix A

**Table A1:** Word pairs used in Experiments 1a and 1b.

Lithuanian	English
Akis	eye
Batas	shoe
Burna	mouth
Daina	song
Gele	flower
Karalius	king
Lova	bed
Medis	tree
Mesa	meat
Mokykla	school
Nafta	oil
Namas	house
Palaidine	shirt
Pliazas	beach
Pomidoras	tomato
Pupa	bean
Purvas	dirt
Sausainis	cookie
Sesuo	sister
Smegenys	brain
Tiltas	bridge
Upe	river
Vejas	wind
Zele	jelly
